# Large urban–rural disparity in the severity of two-week illness: updated results based on the first health service survey of Hunan Province, China

**DOI:** 10.1186/s12939-016-0330-z

**Published:** 2016-02-29

**Authors:** Danping Tian, Li Sun, Lingling Zhang, Lin Zhang, Wei Zhang, Li Li, Xin Deng, Peishan Ning, Xunjie Cheng, Jing Deng, Guoqing Hu

**Affiliations:** Department of Medical Records and Health Statistics, Hunan Children’s Hospital, Changsha, China; The First Affiliated Hospital of Zhengzhou University, Zhengzhou, China; Department of Public Health Sciences, Clemson University, Clemson, SC USA; Department of Epidemiology and Health Statistics, Xiangya School of Public Health, Central South University, 110 Xiangya Road, Changsha, 410078 China

**Keywords:** Two-week illness, Prevalence, Urban areas, Rural areas, Severity

## Abstract

**Background:**

To examine urban–rural differences in the severity of non-fatal disease and injury using the latest household interview survey data of Hunan Province, China.

**Methods:**

Two-week illness data were from the first provincial health household interview survey of Hunan in 2013. The proportion of patients being bedridden, the average days of being bedridden and the average off-work days were calculated to measure the severity of two-week illness. Rao-Scott-adjusted chi-square test was performed to examine the significance of two-week illness severity differences from demographic variables. Multiple logistic regression and linear regression were used to control for sex, age and household income.

**Results:**

The two-week illness prevalence was 22.8 % in Hunan province. Despite similar two-week ill prevalence rates between urban areas and rural areas (23.0 % vs. 22.8 %), rural residents had higher proportions of being bedridden and of being off work than urban residents after controlling for sex, age and household income, with adjusted odds ratios of 3.4 and 6.9, respectively. Similarly, the average days of being bedridden and of being off work in rural residents were 0.45 days and 1.61 days longer than in urban residents after controlling for demographic variables, respectively.

**Conclusion:**

The recent data shows that two-week illness in rural residents is more serious than urban residents in Hunan Province, China in spite of very similar two-week prevalence rates. The neglected urban–rural disparities in the severity of two-week illness deserve the attention of health policy-makers and researchers.

## Background

Urban–rural health disparities are a global health concern [1]. Even in the United States, large differences were reported in average days of being bedridden and of being off work that were caused by two-week illness between metropolitan statistical areas [2]. Unfortunately, very limited urban–rural disparity data are available regarding urban–rural gaps in the severity of non-fatal diseases and injuries for China, the largest low- and middle-income country in the world, despite that the urban–rural disparity has been documented for injury mortality [3], morbidity [4] and hypertension prevalence [5].

Currently, two-week illness causes a huge number of days of being bedridden and of being off work in China. The first four national health service surveys of China that were conducted in 1993, 1998, 2003 and 2008 have revealed higher severity in two-week illness in rural residents than in urban residents [6, 7]. Extensive efforts and resources have been made to reduce the urban–rural health differences in China during the past decade. Since the implementation of new health reforms in 2009, the central government of China has established the basic medical insurance system nationwide and has initiated the basic public health program at the community level that covers both urban and rural residents [8]. To our knowledge, the recent urban–rural health disparity has not reported since the implementation of these projects in China. Using data from the first health service survey of Hunan Province, China that was completed in 2013, we reported updated urban–rural disparity in the prevalence and severity of two-week illness related to China.

## Methods

### Data source

Data came from the first health service interview survey of Hunan Province, China in 2013. This survey adopted multi-stage random sampling to include 24 282 inhabitants of 8400 households from 7 urban areas and 7 rural areas. During August 2013, data were collected through face-to-face household interviews by trained personnel. This survey focused on health care need and utilization of respondents.

The survey was organized by the Provincial Health and Family Planning Commission of Hunan (former Health Bureau of Hunan). A group of experts inspected the field implementation of household interviews at all 14 sample counties (1–2 days per county) to identify the problems in face-to-face interviews and provide solutions.

During the survey, the interviewers explained the purpose and confidentiality of the survey to interviewees before inviting them to voluntarily participate in this study [9, 10]. Data were entered and checked using the standardized dataset and procedure provided by the National Health and Family Planning Commission of China. The data analysis was de-identified and was approved by the medical ethnic committee of Central South University.

### Outcome variables

A two-week illness was defined if the respondents had any of the following three circumstances in the prior two weeks when interviewed: (1) pay visits to a doctor; (2) receive medical treatment for the illness or injury (such as taking medicines or using auxiliary therapy, therapeutic massage, and hot compress, among others); or (3) being bedridden or being off work due to illness (including obvious abnormal depression and loss of appetite in elderly people and unusual crying in infants) [10].

We calculated average days of being bedridden and of being off work, and proportion of residents of being bedridden and of being off work in the last two weeks to measure the severity of two-week illness. It must be noted that “being bedridden” applied to all age groups while “being off work” applied to merely employed respondents aged 15 years and older.

In addition, we calculated two-week illness prevalence, which was defined as “number of respondents who had two-week illness divided by total number of respondents × 100 %”.

### Independent variables

Based on available data and related publications [6, 7], we selected sex, age, and household income as covariates to examine the impact of location of residence (urban–rural) on two-week illness severity. We divided age into five groups: 0–4 years, 5–24 years, 25–44 years, 45–64 years, and ≥65 years. Households were equally divided into five categories based on household income per capita in the last 12 months for urban areas and rural areas, separately: lowest (urban, <6667 Yuan; rural, <3334 Yuan); low (urban, 6667–9999 Yuan; rural, 3334–4999 Yuan); average (urban, 10000–14999 Yuan; rural, 5 000–7 499 Yuan); high (urban, 15000–23999 Yuan; rural, 7 500–9 999 Yuan); and highest (urban, ≥24 000 Yuan; rural, ≥10 000 Yuan).

### Statistical analysis

Multi-stage sampling weights were applied to data analysis. The Rao-Scott-adjusted chi-square test was performed to examine the significance of differences in two-week illness prevalence rates from demographic variables [11]. Multivariate logistic regression and multiple linear regression were performed to examine urban–rural differences in severity indicators of two-week illness after controlling for sex, age, and household income per capita. Data analysis was completed using the PROC SURVEYFREQ procedure and the PROC SURVEYLOGISTIC procedure of SAS9.1 statistical software [12]. “*P* < 0.05” was considered statistically significant.

## Results

In total, 24 282 of 24 286 residents completed face-to-face interview were required, with a high respondent rate of 99.98 %. The prevalence of two-week illness was 22.8 % in Hunan Province in 2013, 95 % confidence interval: 16.3 %–29.4 % (Table 1). Urban residents had a very similar two-week illness prevalence to rural residents (23.0 % vs. 22.8 %), *P* > 0.05. Except for insignificant differences between age groups, differences from sex and household income per capita were statistically insignificant, *P* > 0.05.

**Table 1:** Weighted proportion of residents having two-week illness by location, sex, age, and household income (Hunan Province of China, 2013)

Demographic variable	*n*	Patients	Weighted proportion (%)		Odds ratio (OR)
			Proportion	95 % CI	
Total	24282	5465	22.8	(16.3, 29.4)	
Location					
Urban	11966	2870	23.0	(18.6, 27.5)	Reference
Rural	12316	2595	22.8	(14.5, 31.1)	0.99
Sex					
Male	12184	2576	21.7	(14.3, 29.1)	Reference
Female	12098	2889	24.0	(18.0, 29.9)	1.13
Age ^*^					
0–4 years	1660	126	5.5	(1.0, 9.9)	Reference
5–24 years	4399	155	3.8	(0.5, 7.0)	0.68^*^
25–44 years	5760	512	10.4	(6.2, 14.7)	2.00^*^
45–64 years	8644	2528	31.5	(22.1, 41.0)	7.32^*^
≥65 years	3819	2144	54.3	(40.7, 67.9)	16.22^*^
Household income per capita^a^					
Lowest	4779	1140	27.2	(20.6, 33.9)	Reference
Low	3601	841	23.2	(15.2, 31.2)	0.82
Average	5480	1154	20.9	(16.0, 25.8)	0.72
High	4338	994	21.9	(14.8, 29.0)	0.77
Highest	6011	1308	22.4	(13.3, 31.4)	0.78

^a^Households were equally divided into five categories in urban areas and in rural areas based on household income per capita after eliminating missing values: lowest (urban, <6 667 Yuan; rural, <3 334 Yuan); low (urban, 6 667–9 999 Yuan; rural, 3 334–4 999 Yuan); average (urban, 10 000–14 999 Yuan; rural, 5 000–7 499 Yuan); high (urban, 15 000–23 999 Yuan; rural, 7 500–9 999 Yuan); and highest (urban, ≥24 000 Yuan; rural, ≥10 000 Yuan)

^*^ *P* < 0.05

The proportions of rural residents of being bedridden and of being off work were much higher than those of urban residents after adjusting for sex, age group, and household per capita, accordingly with adjusted odds ratio (OR) of 3.4 and 6.9 (Table 2).

**Table 2:** Adjusted urban–rural differences in weighted proportions of residents being bedridden and being off-work in the prior two weeks based on multivariate logistic regression (Hunan Province of China, 2013)

Severity	Location	*n*	Weighted proportion (%)		Adjusted OR (95 % CI) ^c^
			Proportion	95 % CI	
Being bedridden ^a^	Urban	2870	6.4	(4.3, 8.4)	Reference
	Rural	2595	18.1	(11.1, 25.2)	3.4 (2.0, 5.9)^*^
Being off work ^b^	Urban	653	6.7	(3.5, 10.0)	Reference
	Rural	1563	32.4	(19.9, 45.0)	6.9(3.5, 13.6)^*^

^a^Data analysis applied to all respondents

^b^Data analysis applied to only employed respondents aged 15 years and older

^c^Adjusted odds ratio (OR) after controlling for sex, age, and household income per capita based on multiple logistic regression

*95 % CI* 95 % confidence interval

^*^ *P* < 0.05

After controlling for sex, age, and household income per capita, the mean days of being bedridden and of being off work caused by two-week illness were 0.45 days and 1.61 days longer in rural residents than in urban residents, respectively (Table 3).

**Table 3:** Adjusted urban–rural differences in average days of being bedridden and being off-work in the prior two weeks based on multiple linear regression (Hunan Province of China, 2013)

Severity indicators	Location	*n*	Average days		*b* (*s*_*b*_) ^c^	*P*
			Mean	95 % CI		
Being bedridden ^a^	Urban	2870	0.59	(0.38, 0.79)	Reference	
	Rural	2595	0.95	(0.71, 1.19)	0.45 (0.13)	<0.01^*^
Being off work ^b^	Urban	653	0.63	(0.32, 0.93)	Reference	
	Rural	1563	2.15	(0.21, 4.08)	1.61 (0.85)	0.08

^a^Data analysis applied to allrespondents

^b^Data analysis applied to only employed respondents aged 15 years and older

^c^partial regression coefficients after controlling for sex, age, and household income per capita based on multiple linear regression

^*^ *P* < 0.05

## Discussion

Based on the latest population-based survey data, we observe large urban–rural disparities in two-week illness severity in Hunan Province of China, in spite of very similar prevalence rates between urban residents and rural residents. The huge urban–rural gaps remain even after sex, age group, and household income per capita are controlled.

The results are consistent with those of early studies [6, 7, 13]. Compared with the previously reported studies related to China that merely reported the results of univariate analysis, we adjusted the impact of covariates such as sex, age group, and household income per capita in statistical analysis in this study, providing more reliable results.

Although highly similar two-week prevalence rates between urban residents and rural residents are encouraging for health disparity reduction in China, our findings reveal large urban–rural gaps in two-week illness severity. Considering high similarity between Hunan Province and many other provinces of China in social and economic development, our results reflect the status of whole country to a great extent. The results indicate that rural residents suffer from more serious two-week illness compared to urban residents currently.

The large differences may be due to differences in health literacy, healthy behaviors and exposure to serious diseases and injuries of residents, health resource allocation, and health care service capacity between urban areas and rural areas. Compared to urban residents, rural residents have relatively poorer public health services and lower health literacy [14]. A study of 876 residents in Hunan Province reported that rural residents had more unhealthy behaviors/habits [15].

In China, health costs and per capita health costs in urban areas were 3.1 times and 2.8 times those in rural areas in 2012, respectively [16]. According to the statistics of Bureau of Statistics of Hunan Province in 2013, health care expenditure was 4 times higher in urban areas than in rural areas [17]. This imbalance of health financing results in fewer medical institutions, medical equipments, and medical professionals in rural areas. Compared to rural areas, urban areas have larger number of expensive equipment (>10,000 Chinese Yuan) [16]. According to *the Chinese Health Statistics Yearbook of 2013*, the number of hospital beds (per thousand people) in urban areas was 2.2 times higher than that in rural areas [16]; the urban–rural difference was 2.5 times in Hunan province [16]. The number of health technicians, practitioners, and registered nurses (per thousand people) in China’s urban areas are 2.5 times, 3.0 times, and 3.0 times higher than rural areas in 2012 [16]; the corresponding differences were 2.8 times, 3.4 times, and 4.1 times in Hunan Province, respectively [16].

Our study underlines that large urban–rural health disparity exists although two-week prevalence rates of urban residents and rural residents have become similar through the efforts of many years. This further suggests more efforts should be made in addition to ongoing work to narrow urban–rural health gaps, such as increasing health investment (including funding, health care facilities, and health care professionals), raising health awareness and changing unhealthy behaviors of residents in rural areas. To be concrete, China should consider improving health care service in rural areas by increasing primary care coverage and enhancing the quality of ongoing basic public health service program.

The study had limitations. First, we did not consider urban–rural disparity in two-week illness severity for specific diseases and injuries due to the limits of sample size. Second, for the same reason, we cannot analyze urban–rural differences in other non-fatal indicators, such as incidence rate and hospitalization rate. Third, we did not include the type of employment in our data analysis because of lack of high-quality and detailed data. Due to the absence of employment information, we could not examine the effect of the type of employment on the urban–rural disparity in the severity of two-week illness. In addition, we cannot examine changes in two-week illness severity due to lack of longitudinal data.

## Conclusion

In conclusion, large urban–rural gaps were observed in two-week illness severity in Hunan Province of China in 2013. Despite similar two-week prevalence rates, the large severity disparity suggests that more efforts should be made by the central and local government of China to improve the health of rural residents. This study provides real-world evidence with health policy makers to support the increase in health care investment in rural area and strengthening the construction of rural health services, in order to reduce the urban–rural health differences in China.

### Note about data access

The raw data of first health service survey of Hunan province were accessed under the contract between School of Public Health, Central South University and the Provincial Health and Family Planning Commission of Hunan. Please contact the corresponding author (Guoqing Hu) if there is any concern related to the results of this paper.

## Abbreviations

OR: odds ratio
